# Genetics and Epigenetics of Glioblastoma: Applications and Overall Incidence of IDH1 Mutation

**DOI:** 10.3389/fonc.2016.00016

**Published:** 2016-01-29

**Authors:** Aizhen Liu, Chunfeng Hou, Hongfang Chen, Xuan Zong, Peijun Zong

**Affiliations:** ^1^Department of Oncology, Yidu Central Hospital, Jinan, China; ^2^Department of Oncology Nursing, Yidu Central Hospital, Jinan, China; ^3^Department of Oncology, Shandong University School of Medicine, Jinan, China

**Keywords:** glioblastoma, genetics, epigenetics, IDH1 mutation

## Abstract

Glioblastoma is the most fatal brain cancer found in humans. Patients suffering from glioblastoma have a dismal prognosis, with a median survival of 15 months. The tumor may develop rapidly *de novo* in older patients or through progression from anaplastic astrocytomas in younger patients if glioblastoma is primary or secondary, respectively. During the past decade, significant advances have been made in the understanding of processes leading to glioblastoma, and several important genetic defects that appear to be important for the development and progression of this tumor have been identified. Particularly, the discovery of recurrent mutations in the *isocitrate dehydrogenase 1* (*IDH1*) *gene* has shed new light on the molecular landscape in glioblastoma. Indeed, emerging research on the consequences of mutant IDH1 protein expression suggests that its neomorphic enzymatic activity catalyzing the production of the oncometabolite 2-hydroxyglutarate influences a range of cellular programs that affect the epigenome and contribute to glioblastoma development. One of the exciting observations is the presence of IDH1 mutation in the vast majority of secondary glioblastoma, while it is almost absent in primary glioblastoma. Growing data indicate that this particular mutation has clinical and prognostic importance and will become a critical early distinction in diagnosis of glioblastoma.

## Introduction

Glioblastoma is the most common brain malignancy and one of the most aggressive cancers found in humans. It is divided into primary and secondary types. Both are histologically identical, so clinical features are used to distinguish them. Primary glioblastoma manifests rapidly *de novo* without recognizable precursor lesions, and is by far the more common, accounting for 80% of cases. It predominates in elderly patients and is characterized by rapid progression and short survival time. Secondary glioblastoma evolves from lower-grade gliomas, such as grade II diffuse astrocytoma or grade II anaplastic astrocytoma, and is typically seen in younger patients. It can be diagnosed with clinical or histological evidence (1). Infiltrating glioblastomas are incurable with current treatment modalities that include surgery, radiation, and chemotherapy. For newly diagnosed patients, the standard treatment is total removal, if possible, followed by the combination of chemotherapy and radiotherapy (2, 3). However, despite this maximum treatment, the prognosis of patients remains dismal with a median survival of 15 months only (2). When compared with other malignancies, there have only been small improvements in the prognosis of glioblastoma patients over recent decades. Nevertheless, understanding of the molecular pathogenesis of glioblastoma has greatly increased and is beginning to match that of other types of cancers. Several important genetic alterations have been known for some time, but new technologies have allowed much deep genetic and epigenetic analysis of large numbers of glioblastoma samples, leading to a number of novel discoveries. One of the most exciting and clinically relevant observations was the discovery that a high percentage of secondary glioblastomas and a very small percentage of primary glioblastoma harbor mutations in the *isocitrate dehydrogenase 1* (*IDH1*) gene. This stunning and unexpected discovery that holds clear clinical implications has led to new insights into glioblastoma biology. Indeed, IDH1 mutation results in gain of function to catalyze the production of hydroxyglutarate (2-HG) (4), a possible oncometabolite that is thought to influence a range of cellular programs involved in epigenetic control and various processes leading to tumor development. Here, we review translational applications of this mutation as well as its incidence in glioblastoma and other cancers. In addition, we highlight importance of epigenetic changes that may need to be considered in future diagnostics and therapy for glioblastoma. Initially, the review presents a brief overview on mutations commonly found in glioblastoma, and prunes the consequences of IDH1 mutation on glioblastoma biology to better understand the potential role of this particular mutation in the development of this tumor.

## Common Mutations in Glioblastoma

Although glioblastoma-specific mutations are seen, mutations in common cancer genes, such as TP53 and PTEN, are very frequent in glioblastomas, but are not of prognostic importance (Table 1) (5, 6). EGFR point mutations have also been identified in glioblastoma. The *EGFRvIII* mutant lacks 267 amino acids in the extracellular part, resulting in a constitutively activated receptor that no longer requires its ligand EGF to signal downstream (5). Although mutations in certain cancer genes, such as *BRAF* and the *RAS* genes, have rarely been observed in glioblastoma, inactivating mutations and deletions have been identified in their inhibitory tumor-suppressor gene *NF1* (5). Mutations in *PIK3CA* and *PIK3R1* genes, coding, respectively, for the PI3K catalytic subunit p110α and regulatory subunit P85α, have also been described (5, 6).

**Table 1:** **Genes commonly mutated in glioblastoma**.

Gene symbol	Gene name	Function of encoded protein	Point of mutation (%)
EGFR	Epidermal growth factor receptor	Regulator of cell signaling involved in cell proliferation and survival	14–15
ERBB2	V-erb-b2-erythroblastic leukemia viral oncogene homolog 2	Regulator of cell signaling involved in cell proliferation and survival	0–7
IDH1	Isocitrate dehydrogenase 1	NADPH production	12–20
NF1	Neurofibromin 1	Regulator of cell signaling involved in cell proliferation and survival	15–17
PIK3CA	Phosphoinositide-3-kinase catalytic alpha	Regulator of cell signaling involved in cell proliferation and survival	7–10
PIK3R1	Phosphoinositide-3-kinase regulatory 1	Regulator of cell signaling involved in cell proliferation and survival	7–8
PTEN	Phosphatase and tensin homolog	Regulator of cell signaling involved in cell proliferation and survival	24–37
PTPRD	Protein tyrosine phosphatase receptor type D	Regulator of cell signaling involved in cell proliferation and survival	0–6
RB1	Retinoblastoma 1	Regulator of cell cycle	8–13
TP53	Tumor protein p53	Apoptosis	31–38

An interesting gene found to contain mutations in glioblastoma is *IDH1*, which encodes IDH1 and is involved in energy metabolism. This gene shows differential expression between primary and secondary glioblastoma, while PTEN loss, EGFR amplification, and loss of heterozygosity (LOH) of chromosome 10 are associated with primary glioblastoma, and ATRX mutation, loss of p53, and LOH of chromosome 19 are common in secondary glioblastoma. The IDH1 mutation predicts secondary glioblastoma better than these other mutations predict their respective glioblastoma subtype. Indeed, IDH1 mutations have been predominantly identified in secondary glioblastoma and low-grade gliomas, with mutations in more than 70% of cases (5, 6). They are found only sporadically in primary glioblastoma (5, 6). Because patients with IDH1-mutated primary glioblastoma are generally younger and have longer median survival and wild-type EGFR, which are characteristics of secondary glioblastoma, it is hypothesized that these are in fact secondary glioblastomas for which no histological evidence of evolution from a less malignant glioma is found. Therefore, IDH1 mutation could be used to differentiate primary from secondary glioblastoma.

## Biological Consequences of IDH1 Mutation

Isocitrate dehydrogenase 1 is a metabolic enzyme that converts isocitrate into α-ketoglutarate (α-KG) via oxidative decarboxylation using NADP+ as an electron acceptor, and producing NADPH (7). While NADPH is thought to be important for limiting cellular oxidative damage and for lipid biosynthesis, α-KG is considered as an essential intermediate in the Kreb’s cycle. Under hypoxia conditions, IDH1 catalyzes the inverse reaction, converting α-KG to isocitrate which can in turn be converted to acetyl-CoA for lipid metabolism and many biochemical reactions.

**Figure 1:**
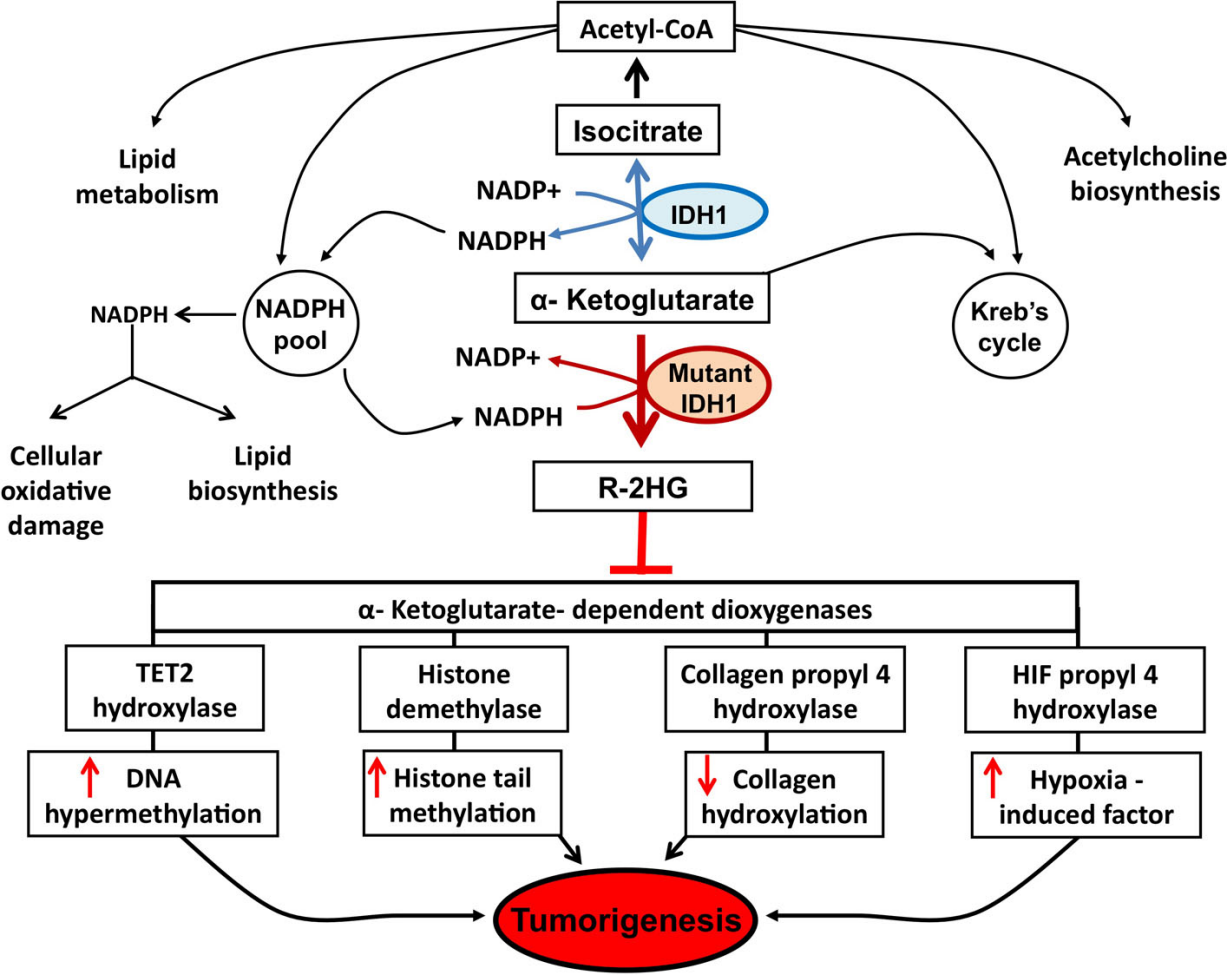
**Potential mechanisms implicated in tumor formation induced by IDH1 mutation**. A number of potential mechanisms have been proposed to explain how R-2-HG produced by mutant IDH1 enzyme promotes tumor formation. Epigenetic modification, via inhibition of α-KG-dependent dioxygenases leading to DNA and histone hypermethylation, has been at the forefront of research efforts. Specifically, the inhibition of tet methylcytosine dioxygenase 2 (TET2) leads to increased DNA hypermethylation and the inhibition of histone demethylases leads to increased histone tail methylation. Additional mechanisms include inhibition of several groups of prolyl hydroxylases, leading to hypoxia-induced factor (HIF) activation and alterations in collagen formation.

Since 2008, with the discovery of recurrent mutations in the IDH1 coding gene by the Vogelstein group analyzing the DNA sequence of the glioblastoma genome (6), substantial progress has been made to understand how such genetic modification leads IDH1 to play a role in the tumorigenesis. The major finding was the discovery that the IDH1 mutation is a gain-of-function mutation, conferring neomorphic activity to IDH1. Initially, a pivotal study profiling IDH1 wild-type and mutant glioblastoma cells with liquid chromatography–mass spectrometry has reported high levels of the metabolite 2-HG in mutant cells (4). Then, it has been demonstrated that mutant IDH1 catalyzes the reduction of α-KG to the R-enantiomer of the metabolite 2-HG (R-2-HG) (8). Thus, rather than catalyze the NADP+-dependent production of α-KG, mutant IDH1 catalyzes the NADPH-dependent reduction of α-KG to produce only the R-enantiomer of 2-HG, indicating a gain of neomorphic function. Specifically, authors have demonstrated that the mutation reduces the affinity of the IDH1 active site for isocitrate while concomitantly increasing it for NADPH and α-KG (8). Reduced affinity for isocitrate occurs as a result of alterations to a binding site residue that forms hydrogen bonds between the alpha and beta groups of isocitrate (8). Consequently, the conversion of α-KG is favorized, but rather than isocitrate, the mutant enzyme converts α-KG into R-2-HG, an oncometabolite that promotes tumorigenesis through inhibition of α-KG-dependent (Figure 1).

## Incidence of *IDH1* Mutation in Glioblastoma and Other Cancers

Extensive genomic profiling has revealed that about 90% of IDH1 mutations involve exon 4 at codon 132, replacing arginine with histidine (R132H). Of the remaining 10% of IDH1 mutations, 4.7% are due to arginine being replaced with cysteine (R132C), 2.1% with glycine (R132G), 1.7% with serine (R132S), 0.8% with leucine (R132L), and 0.3% with glutamine (R132Q) (9).

Following the first observation of recurrent IDH1 mutation in glioblastoma, several groups have begun to clarify the frequency and distribution of IDH1 mutation across all brain tumors, including gliomas and other subtypes. Data summarized from many studies show that only approximately 5.6% of primary glioblastoma are IDH1 mutant, while more than 76% of secondary glioblastomas carry the IDH1 mutation. The reported rates of IDH1 mutation in lower-grade gliomas are comparable with those of secondary glioblastoma (Table 2) (10).

**Table 2:** **Frequency of IDH1 mutation in various tumors**.

Tumor types	IDH1 mutation frequency (%)
Astrocytoma pilocytic (WHO Grade I)	0.01
Diffuse glioma (WHO Grade II)	76
Astrocytoma anaplastic (WHO Grade III)	62.2
Primary glioblastoma (WHO Grade IV)	5.6
Secondary glioblastoma (WHO Grade IV)	76.4
**Oligodendroglioma**	
WHO Grade II	78.8
WHO Grade III	67.5
**Oligoastrocytoma**	
WHO Grade II	79.8
WHO Grade III	69.7

*CNS, central nervous system; WHO, World Health Organisation; AML, acute myeloid leukemia; d-2-HGA, d-2-hydroxyglutaric aciduria; MDS, myelodysplastic syndrome; MPN, myeloproliferative neoplasms*.

Other brain tumors harboring IDH1 mutations with moderate frequency include gangliogliomas, giant cell glioblastomas, and primitive neuroectodermal tumors, although only small numbers of these tumors have been studied (11).

Isocitrate dehydrogenase mutations are also present in some tumors originating in cells outside of the central nervous system. Indeed, about 7.7% of acute myeloid leukemia (AML) possessed the IDH1 mutation (12), but prevalence rates vary between 15 and 33% if IDH2 are also considered (13). Approximately 50% of d-2-hydroxyglutaric aciduria (d-2-HGA), a rare inherited neurometabolic disorder, has also been found to display *IDH1* mutations (14), as well as 10% of intrahepatic cholangiocarcinomas (15), 5% of myelodysplastic syndrome (MDS), 8.8% of myeloproliferative neoplasms (MPN), and fewer than 10% of secondary AML (14). In their investigation, Amary and colleagues found that nearly 60% of central and periosteal cartilaginous tumors displayed *DH1*mutations (16). The same group has also identified *IDH1* mutation to occur in patients with Ollier disease and Maffucci syndrome (17). The majority of patients exhibited the R132C IDH1 mutation, in contrast to most secondary glioblastoma, which harbor the R132H mutation.

## Translational Applications of Mutant IDH1

In a relatively short time after the discovery of *IDH1* mutation in glioblastoma, a tremendous amount of work has been performed on the clinical relevance of this mutation regarding particularly its applications in the diagnosis, prognosis, and treatment of patients suffering from glioblastoma.

### Diagnostic Applications

The determination of IDH1 mutation status is highly relevant for the diagnosis of primary brain tumors, and strongly supports the differential diagnosis between an anaplastic glioma and a glioblastoma.

Traditionally, IDH1 mutation status was detected through classic Sanger sequencing and polymerase chain reaction (PCR). Although this has the clear advantage of being able to detect non-R132H mutation, it is time consuming and requires there to be at least 20% mutant allele frequency within the tissue specimen for reliable detection (18). Pyrosequencing is an alternative that allows for rapid high-throughput analysis of IDH1 mutation, and has demonstrated an advantage over classic Sanger sequencing in that it can detect mutated allele frequencies down to 5% (19). Derived cleaved amplified polymorphic sequence analysis is another alternative to DNA sequencing that uses mismatched primers for specific mutations, which, following PCR amplification will create differing restriction endonuclease sites dependent on the presence of the mutation (20). The advantage of this technique is that it uses supplies commonly found in most laboratories, obviating expensive sequencing equipment. However, unlike sequencing, the method is limited in that it can only detect mutations being queried. Other PCR-based techniques include coamplification at lower temperature (COLD) PCR with high-resolution melting (HRM) and real-time PCR, and post-PCR fluorescent melting curve analysis (FMCA). Through COLD PCR combined with HRM, Boisselier and colleagues were able to detect mutant allele concentrations of 0.25% in a span of only 3 h (21). However, because the technique requires the new mutation to have a melting temperature (Tm) that is lower than IDH1 wild type, it theoretically may not be able to detect R132G mutation. Regarding real-time PCR with post-PCR FMCA, this technique was shown to be more sensitive than Sanger sequencing with detection rate of as little as 10% mutant DNA and a processing time of 80 min.

Other technologies currently in use to detect mutations in the IDH1 gene include SNaPshot (22) and Oncomap (23), both of which can be used with paraffin-embedded tissue, using base pair extension that results in an allele-specific probe that is read out by either fluorescence detection (SNaPshot) or mass spectrometry (Oncomap). In addition to these approaches, Boisselier and colleagues demonstrated evidence of principle in detection of *IDH1* mutations from the plasma of patients with mutant glioma (24). Although the potential application for monitoring disease non-invasively is compelling, the sensitivity attained using this assay was 60%. Further work on the nature of circulating tumor material will be necessary to determine whether it will be possible to monitor the IDH1 mutation status in the peripheral blood of all patients with mutant gliomas.

Recently, a new technique called amplification-refractory mutation system (ARMS) has been developed (25) to identify R140Q mutations in *IDH2* and such novel methods can be of great help in detecting IDH1 mutations. Recently, a novel strategy of PCR clamping was employed for qualitative detection of seven different mutations in IDH1 and five mutations in IDH2 in a single PCR assay (26).

A monoclonal antibody specific for the *IDH-R132H* mutation (mIDH1R132H), developed by Capper’s group, recognizes the mutant protein with a high degree of sensitivity and specificity (27). Using this antibody, 90% of IDH1 mutation can be detected on paraffin sections, and it is recommended to test the remaining 10% by sequencing. Nevertheless, proponents of immunohistochemistry-based antibody staining argue that the use of mIDH1R132H antibody to identify IDH1 mutation may even be superior to direct sequencing because there are reported cases in which this antibody detects mutations missed by direct sequencing (28).

Beyond determination of *IDH1* mutation status, the histopathological utility of the *mIDH1R132H* antibody has extended to additional clinical scenarios such as the discrimination between diffuse astrocytoma and reactive astrocytosis when combined with a panel of key molecular feature (29). Indeed, seeing that *IDH1* mutations are specific to gliomas, this antibody can be used to help differentiate between diffuse gliomas and areas of reactive gliosis. Accordingly, the detection of positive cells by IDH1-R132H immounohistochemistry allows a clear and safe separation between low-grade glioma and reactive gliosis, and clearly supports the diagnosis of a diffusely infiltrating glioma as well as the differential diagnosis between an anaplastic glioma and a glioblastoma. This approach allows narrowing down the possible diagnosis to the group of diffusely infiltrating of WHO grade II and III and secondary glioblastoma and to a certain extent to primary glioblastoma. IDH1-R132H immounohistochemistry has also been shown to be effective in separating out oligodendrogliomas from several other similar entities, such as central neurocytomas, tanycytic ependymomas, and pilocytic astrocytomas (27).

Another area of investigation has focused on the detection of the 2-HG metabolite rather than on the specific sequence of the *IDH1* gene or protein product. In theory, sensitive and specific detection of 2-HG is sequence independent in that 2-HG should be present regardless of the type of mutation in *IDH1* or *IDH2*. High levels of 2-HG have been detected in *ex vivo* tissue samples by using two approaches. In the first approach, combination gas or liquid chromatography/mass spectrometry was used to identify 2-HG in frozen or paraffin-embedded glioma tissue. However, these extraction-based approaches do not preserve the integrity of the sampled tissue. In the second approach, proton high-resolution magic angle spinning (HRMAS) MRS is used to determine the metabolic profiles in *ex vivo* tissue. This technique does not require alteration of tissue samples, and identified 2-HG in *ex vivo* specimens with high sensitivity and specificity. Unlike the case with AML, wherein 2-HG can be detected in the blood of patients with *IDH*-mutant AML (30), its presence in peripheral blood is similar between patients with *IDH1*-mutant and wild-type tumors (31).

Detection of 2-HG by MRS represents a completed non-invasive method with which to determine the presence of *IDH* mutations in gliomas, irrespective of the sequence of the mutation or the mutation maps to *IDH1* or *IDH2*. Importantly, this approach represents the only example in human cancer in which a genomic feature can be identified specifically by using imaging-based metabolic profiling. Prior work completed by MRS-based approach has demonstrated that *IDH*-mutant tumors display characteristic imaging findings. The IDH1-mutant tumors display reduced contrast enhancement, less surrounding edema, cystic components, and often found in the frontal lobe compared with IDH-wild-type tumors (32). Because it is recognized that the spectrum of 2-HG has some overlap with other commonly found metabolites, such as glutamate and glutamine, the methodology for obtaining and analyzing *in vivo* MRS is critical. One study to demonstrate 2-HG detection in glioma by MRS used the acquisition of a point-resolved spectroscopy (PRESS) sequence (33). There was good concordance between sequence-based mutation status and 2-HG detection, although several false positives and false negatives were reported. Additional studies in which spectral-editing analysis (34) or 2D-correlation MRS with spectral editing (35) were used demonstrated that IDH mutation status can be identified non-invasively by MRS techniques with a high levels of sensitivity and specificity.

### Prognostic Implications

Extensive research has been performed to determine the prognostic value of *IDH1* mutations, and a better prognosis has been generally reported in glioblastoma patients carrying an *IDH1* mutation. Studies have shown that *IDH1* mutation convey an improved prognosis with respect to both overall survival and progression-free survival for the rare glioblastoma patients who express this mutation. Indeed, since the publication of the first report on improved survival in glioblastoma patients with mutation by Parsons and colleagues, indicating 45.6 months overall survival in *IDH1*-mutant versus 13.2 months overall survival in *IDH1* wild type (6), numerous groups have been able to replicate similar findings (36–41). Besides improved overall survival, Sanson et al. (37) were able to demonstrate improved progressive-free survival (PFS) as well in their set of patients with glioblastoma, with 55 months PFS in patients with *IDH1* mutation versus 8.8 months PFS in those without this mutation. The analysis was extended to anaplastic (WHO grade III) tumors because many groups were readily able to show an improved overall survival in grade III tumors that harbored the *IDH1* mutation compared with those that did not in both univariate (36) and multivariate analyses (37, 40). With anaplastic astrocytomas, patients harboring *IDH1* mutation had an overall survival of 65 months compared to 20 months for patients without *IDH1* mutation (38). The survival benefit also extended to grade II gliomas, showing a median overall survival of 12.6 years in patients with *IDH1* mutation versus 5.5 years in patients devoid of this mutation. In a prospective analysis, Wick and colleagues found that grade III astrocytomas that possessed the IDH1 mutation were associated with greater PFS regardless of the treatment (42).

In studies pooling low-grade astrocytomas and oligodendrogliomas, the IDH1 mutation status was prognostic for overall and PFS. In primary glioblastoma, IDH1 mutational status has been reported to be the only factor that showed significant association with patient survival times. However, the evidence for low-grade gliomas and the prognostic value of IDH1 mutation is slightly more controversial. Two independent groups found that IDH1 mutations in low-grade gliomas were associated with significant improved overall survival (43, 44), whereas others could not find any significant association (36, 45). Nevertheless, the consistent finding of a more favorable outcome of diffuse gliomas patients with IDH1 mutation implies that IDH1 testing might be useful for prognostic considerations in the clinical setting.

### Predictive Implications

Isocitrate dehydrogenase 1 mutation was correlated with a higher rate of response to up-front temozolomide in low-grade glioma patients. Furthermore, evidence for differential responsiveness to genotoxic therapy of IDH1 mutant versus IDH1 wild-type low-grade gliomas has been provided. Indeed, the presence of IDH1 mutation was associated with favorable progression-free and overall survival in WHO grade II gliomas who received radio- or chemotherapy. Current studies aim to validate and clarify the predictive value of *IDH1* mutation in the different glioma types.

It is still unclear if *IDH1* mutational status is a prognostic indicator or predictive measure of response to treatment. Houillier and colleagues stratified a cohort of low-grade gliomas into three groups based on prognostic factors according to the presence of 1p19q deletion, *IDH1* mutation, or both together (46). They found that each of these factors was an independent predictor of improved clinical outcome in response to treatment with the chemotherapeutic agent temozolomide and that the group of patients with both mutations had the best treatment response. Objective response was in 80% with both mutations, 61% of *IDH1*-mutants without 1p19q deletion, and 17% without either mutation. In a similar fashion, Hartman and colleagues found that, in their cohort of patients receiving adjuvant therapy, *IDH1* mutation status was the single most important predictor of PFS and overall survival. This was not seen in their cohort of patients that did not receive adjuvant therapy (47). These findings support the notion that *IDH1* mutation may be an important predictor to treatment response.

### Treatment Implications

The identification of *IDH1* mutation and the rapid characterization of its protein products present a therapeutic opportunity to treat the *IDH1*-mutant tumors. From a medical standpoint, it will be important to determine whether these mutant tumors would be sensitive to molecules that inhibit the mutant enzyme. Although there are no published studies to date addressing this possibility, one study performed by Jin and colleagues suggested that mutant tumor cells may depend on the continued expression of the mutant enzyme and/or its resulting 2-HG metabolite. In their investigation, the authors showed that a cell line expressing endogenous mutant *IDH1* required its expression for survival and anchorage-independent growth (48), suggesting that pharmacological inhibition of mutant *IDH1* may recapitulate this result. With respect to mutant *IDH1*-mediated biology, this result would also suggest that 2-HG-induced changes, such as global DNA hypermethylation, are either potentially reversible or, if not, are insufficient for tumor maintenance. It may be anticipated that inhibition of this pathway would increase patient survival. Although there has been some discussion of whether it is prudent to inhibit mutant IDH1 because patients with mutant tumors have a better survival than patients with wild-type tumors, it is not expected that inhibiting the mutant enzyme would make mutant tumors behave like their more aggressive wild-type counterparts. The differences in survival most likely stem from the fact that IDH1-mutant and wild-type tumors arise from distinct lineages and ontogenies and, thus, represent entirely different neoplastic disease processes.

## Epigenetic Changes in Glioblastoma

Epigenetic is the mitotically heritable changes in gene expression that are not due to changes in the DNA sequence, and has emerged as hallmark of human cancers. Indeed, aberrant epigenetic mechanisms, such as DNA methylation, histone modifications, chromatin remodeling, or altered non-coding RNA expression, are currently recognized as relevant events in tumor formation (49). Until now, most studies about the epigenetic changes in glioblastoma have focused on DNA methylation, including hypermethylation, gene-specific hypomethylation, and genome-wide hypomethylation (50). The leading mechanism attributed to the observed hypermethylation phenotype in IDH1 mutant involves silencing of the α-KG-dependent DNA modifying enzyme, Tet methylcytosine dioxygenase 2 (TET2) (Figure 1).

Since the first report demonstrating that a subset of glioblastoma exhibits a global decrease in 5-methylcytosine, subsequent follow-up studies have revealed not only that genome-wide or global hypomethylation occurs at a frequency of 80% in primary glioblastoma, but also that the level of hypomethylation varies between glioblastomas, ranging from near normal levels to approximately 50% of normal in about 20% of cases (51). The most severe globally hypomethylated primary glioblastomas are also the most proliferative and display dramatic hypomethylation (22–50% of normal brain) of the tandem repeat satellite 2 (Sat2) located at the juxtacentromeric region of chromosomes 1, 9, and 16, and moderate hypomethylation (71–80%) of the D4Z4 located at the subtelomeric regions of chromosomes 4q35 and 10q26 (51). Moreover, glioblastoma with hypomethylated Sat2 also harbored copy number alterations of adjacent euchromatic sequences, specifically near the pericentromeric region of chromosome 1. These data suggest that one consequence of hypomethylated repetitive sequences in glioblastoma is predisposition to chromosomal breakage and copy number alteration. Although the full consequences of genomic hypomethylation are unknown, murine models of defective imprinting provide evidence for a causal role of DNA methylation alteration in tumorigenesis.

DNA methylation has been shown to play critical roles in the control of gene activity and the architecture of the nucleus of the cell. In humans, DNA methylation occurs in cytosines that precede the guanines to create 5-methylcytosine and these are commonly called dinucleotide CpGs (52). These dinucleotides are not randomly distributed in the genome, but instead are present as CpG-rich regions referred to as CpG islands. Hypermethylation of CpG islands in the promoter regions of tumor-suppressor genes is a major event in the origin of many cancers. In glioblastoma, CpG promoter hypermethylation occurs at genes with diverse functions related to tumorigenesis and tumor progression, including cell cycle regulation (*CDK2A-p16INK4a* and *CDK2B-p15INK4b*) tumor suppression (*RB1, VHL, EMP3, RASSF1A*, and *BLU*), DNA repair [methylguanine DNA methyltransferase (*MGMT*) *and MLH1*], inhibition of apoptosis (DAPK1, TIMP3, CDH1), and genes associated with angiogenesis, regulation of tumor invasion, and drug resistance (53). Many new tumor-suppressor candidates have been identified, including the cell motility regulator testis-derived transcript (*TES*) as well as many polycomb repressor complex 2 (PRC2) target genes (54), and epithelial membrane protein 3 (*EMP3*), a myelin-related gene involved in cell proliferation and cell–cell interaction that is silenced by hypermethylation in glioblastoma (55).

Promoter hypermethylation has been demonstrated to regulate the oncogenic and proliferation-promoting transforming growth factor (TGF)-beta signaling pathway in aggressive, highly proliferative glioblastomas. High levels of TGF-beta signaling are normally associated with poor prognosis. TGF-beta signaling promotes proliferation through the induction of platelet-derived growth factor-beta (*PDGF*-*B)*. However, epigenetic silencing of *PDGF-B* can override the increased proliferative effects of TGF-beta signaling. Specifically, *PDGF-B* promoter hypermethylation prevents *PDGF-B* transcriptional activation by TGF-beta-induced Smad proteins (56). The oncogenic effect of the TGF-beta pathway is, therefore, blocked by epigenetic alteration of one of its targets.

Genes involved in invasion and metastasis can also be affected by promoter hypermethylation in glioblastoma. Approximately 87% of glioblastoma exhibit CpG hypermethylation of the protocadherin-gamma subfamily A11 (*PCDH-gamma-A11*) gene, which is thought to be important in invasion of cancer cells into normal brain parenchyma (57).

The hypermethylation of promoter can also modulate sensitivity to drugs and radiotherapy in glioblastoma. The best known example is the O6-*MGMT* promoter methylation and response to alkylating agents, but there is also the gene suppressor of cytokine signaling 1 (SOCS1), which in some glioblastomas enhanced resistance to ionizing radiation and decreased activation of MAPKs associated with the ERK pathway after transcriptional silencing by hypermethylation (58). This suggests that epigenetic profiling might be one way to categorize glioblastomas and to rationally apply patient-specific therapy.

For certain sets of genes, promoter hypermethylation might not be causal or may not be required for gene silencing in glioblastoma. There is some evidence for deregulation of gene controlling histone modifications in this tumor. The gene encoding *BMI-1*, a member of the polycomb group complex that regulates histone H3K27 methylation, is subject to frequent copy number alterations in both low- and high-grade gliomas (59). Expression of some histone deacetylase (HDAC) proteins is reported to be altered in glioblastoma. Class II and IV HDACs displayed decreased mRNA expression in glioblastoma compared to low-grade astrocytomas and normal brain, and overall histone H3 was more acetylated in glioblastoma (60). Large-scale sequencing of protein-coding genes in glioblastoma revealed mutations in many genes involved in epigenetic regulation, including HDACs, *HDAC2* and *HDAC9*, histone demethylases, *JMD1A* and *JMD1B*, histone methyltransferases, *SET7*, *SETD7*, *MLL*, *MLL4*, and methyl-CpG binding domain protein1 (*MBD1*) (6). These intriguing initial studies suggest that alteration in epigenetic mechanisms could be a major defect in glioblastoma.

## Conclusion

Following the discovery of IDH1 mutation, our understanding of the biochemistry, genetics, and epigenetics as well as the prevalence and pathogenic role of this mutation has grown at a rapid rate, and a tremendous amount of work has been performed on its translational relevance in a relative short time. It is, henceforth, clear that IDH1 status is a major determinant of survival, and many ways have been developed to identify the IDH mutation as well as the oncometabolite 2-HG from clinical samples, using non-invasive procedures. The determination of IDH1 status in glioblastoma will likely be an early step in treatment algorithms for patients suffering from this tumor.

## Author Contributions

The authors collected and analyzed the information and wrote the manuscript.

## Conflict of Interest Statement

The authors declare that the research was conducted in the absence of any commercial or financial relationships that could be construed as a potential conflict of interest.
